# Detection of the Adulteration of Quinine with Salicine

**Published:** 1871-01

**Authors:** 


Detection of the Adulteration of Quinine with Salicine.—Dr. Solenén, says the *American Chemical News*, has comparatively tested the degree of accuracy and sensitiveness of the different tests in use for the detection of the presence of salicine in quinine, which, if made with the view of fraudulent adulteration, will always be at least at the rate of one per cent. of salicine or more, because less will not pay. The author employed three kinds of sulphuric acid, *viz.*, the fuming, pure concentrated acid, free from arsenic and nitric acid; ordinary concentrated sulphuric acid of commerce, containing a trace of nitric acid; and, lastly, sulphuric acid, to which, purposely, nitric acid had been added. A watch-glass having been placed on a sheet of white paper, and a drop or two of the acids above referred to (each in a separate glass) having been poured therein, a few crystals of the alkaloid (sulphate of quinine) were put on the acid; if pure, there is no coloration, but even with one-hundredth of salicine, the two first-named acids caused a distinct red coloration, which did not ensue with the acid containing nitric acid. This latter acid was not even colored by pure salacine.—*Medical and Surgical Reporter.*

